# Supplementary material for: Multi-Domain Touchscreen-Based Cognitive Assessment of C57BL/6J Female Mice Shows Whole-Body Exposure to 56Fe Particle Space Radiation in Maturity Improves Discrimination Learning Yet Impairs Stimulus-Response Rule-Based Habit Learning
Source: Front Behav Neurosci. 2021 Oct 11;15:722780. doi: 10.3389/fnbeh.2021.722780 (PMC8543003; doi:10.3389/fnbeh.2021.722780)
Supplement: Supplementary file 1 [file Table_1.pdf]

Supplementary Table 1. Complete statistical analyses for each figure panel and all results.

Bold text and ***, p<0.05. Italicized text, 0.05<p<0.1. *Magnitudes of partial omega-squared (for RM two-way ANOVA): 0.01 small; 0.06 medium; 0.14 large. N/A not applicable.

Subject	Figure	Home Institute (NSRL run)	n	Mean								Statistics (variables)	Main Effect *p<0.5 **p<0.01 ***p<0.001 ****p<0.0001	F Value	p value	Post hoc Test (Bonferroni)	Effect size (when RM two-way ANOVA p<0.05, partial omega-squared is calculated where ≤0.05 small, ≥0.06 medium, ≥0.14 large)
Weights	2A	CHOP (NSRL 18A)	Sham: 16	Post-IRR (wks)	0	16	22	28	34	40	46	Mixed-effects analysis	time****	F (36, 1067) = 66.05	**p<0.0001**		
				Sham	25.4	29.19	28.53	30.26	31.18	31.09	36.52						
			Frac 20 cGy: 16	Frac 20 cGy	24.92	29.91	28.83	29.94	30.92	30.76	38.3		treatment	F (1, 30) = 0.02450	p=0.8767	Sham vs. 56Fe: all p>0.05	
													interaction**	F (36, 1067) = 1.586	**p=0.0161**		
TS Locomotion	2B	CHOP (NSRL 18A)	Sham: 16	LM								Unpaired t-test	NA	NA	p=0.6979	NA	
				Sham	199.3 ± 15.75												
			Frac 20 cGy: 16	Frac 20 cGy	191.9 ± 10.29												
General Touchscreen Training w/six windows	2C	CHOP (NSRL 18A)	Sham: 15	General TS training								Two-way RM ANOVA	interaction	F (5, 145) = 1.035	p=0.3992	NA	
				Training Stage	HAB1	HAB2	IT	MT	MI	PI			training stage****	F (5, 145) = 59.27	**p<0.0001**		0.62
			Frac 20 cGy: 16	Sham	1	1	2.2	5.267	1	13.93			treatment	F (1, 29) = 2.511	p=0.1239		
				Frac 20 cGy	1	1	3.438	5.938	1.125	17.94			subject	F (29, 145) = 1.048	p=0.4105		
Punish incorrect Session length	2D	CHOP (NSRL 18A)	Sham: 15	Session length (s)								Two-way RM ANOVA	interaction	F (1, 29) = 2.345	p=0.1365	Sham vs. 56Fe: *a'* p=0.0228	
				Session	First	Last							session	F (1, 29) = 0.5688	p=0.4568		
				Sham	1055	1217							treatment*	F (1, 29) = 4.532	**p=0.0419**		0.07
			Frac 20 cGy: 16	Frac 20 cGy	1340	1285							subject	F (29, 29) = 1.355	p=0.2092		
Punish incorrect Trial Number	2E	CHOP (NSRL 18A)	Sham: 15	Trial #								Two-way RM ANOVA	interaction	F (1, 29) = 3.742	p=0.0629	NA	
				Session	First	Last							session	F (1, 29) = 3.742	p=0.0629		
				Sham	30	30							treatment	F (1, 29) = 3.742	p=0.0629		
			Frac 20 cGy: 16	Frac 20 cGy	29	30							subject	F (29, 29) = 1.000	p=0.5000		
Punish incorrect Percent correct	2F	CHOP (NSRL 18A)	Sham: 15	% correct								Two-way RM ANOVA	interaction	F (1, 29) = 0.01728	p=0.8963	NA	
				Session	First	Last							session****	F (1, 29) = 149.5	**p<0.0001**		0.69
				Sham	43.11	83.33							treatment	F (1, 29) = 0.5868	p=0.4499		
			Frac 20 cGy: 16	Frac 20 cGy	40.78	80.15							subject	F (29, 29) = 1.225	p=0.2941		
Punish incorrect ITI Touch	2G	CHOP (NSRL 18A)	Sham: 15	ITI Touch								Two-way RM ANOVA	interaction	F (1, 29) = 1.138	p=0.2949	NA	
				Session	First	Last							session****	F (1, 29) = 49.58	**p<0.0001**		0.44
				Sham	22.4	7.133							treatment	F (1, 29) = 0.3334	p=0.5681		
			Frac 20 cGy: 16	Frac 20 cGy	21.5	10.25							subject	F (29, 29) = 1.039	p=0.4591		
Punish incorrect Correct touch latency	2H	CHOP (NSRL 18A)	Sham: 15	Correct touch latency (s)								Two-way RM ANOVA	interaction	F (1, 29) = 1.127	p=0.2971	NA	
				Session	First	Last							session****	F (1, 29) = 43.99	**p<0.0001**		0.38
				Sham	10.5	5.71							treatment	F (1, 29) = 0.2134	p=0.6476		
			Frac 20 cGy: 16	Frac 20 cGy	10.95	4.336							subject	F (29, 29) = 1.349	p=0.2127		
Punish incorrect Correct left touch latency	2I	CHOP (NSRL 18A)	Sham: 15	Correct left touch latency (s)								Two-way RM ANOVA	interaction	F (1, 29) = 0.001474	p=0.9696	NA	
				Session	First	Last							session***	F (1, 29) = 16.07	**p=0.0004**		0.17
				Sham	10.75	6.398							treatment	F (1, 29) = 2.078	p=0.1601		
			Frac 20 cGy: 16	Frac 20 cGy	8.846	4.572							subject	F (29, 29) = 1.448	p=0.1622		
Punish incorrect Correct right touch latency	2J	CHOP (NSRL 18A)	Sham: 15	Correct right touch latency (s)								Two-way RM ANOVA	interaction	F (1, 29) = 0.005288	p=0.9425	NA	
				Session	First	Last							session***	F (1, 29) = 15.34	**p=0.0005**		0.17
				Sham	9.364	5.117							treatment	F (1, 29) = 2.961	p=0.0960		
			Frac 20 cGy: 16	Frac 20 cGy	7.384	2.976							subject	F (29, 29) = 1.174	p=0.3345		
Punish incorrect Blank touch latency	2K	CHOP (NSRL 18A)	Sham: 15	Blank touch latency (s)								Two-way RM ANOVA	interaction	F (1, 29) = 3.234	p=0.0825	Sham vs. 56Fe: *a'* p=0.0203	
				Session	First	Last							session**	F (1, 29) = 9.015	**p=0.0055**		0.12
				Sham	11.65	10.13							treatment*	F (1, 29) = 3.842	**p=0.05**		0.05
			Frac 20 cGy: 16	Frac 20 cGy	11.4	5.338							subject	F (29, 29) = 1.035	p=0.4635		
Punish incorrect Reward latency	2L	CHOP (NSRL 18A)	Sham: 15	Reward latency (s)								Two-way RM ANOVA	interaction*	F (1, 29) = 5.175	**p=0.0305**	Sham vs. 56Fe: all p>0.05	0.07
				Session	First	Last							session****	F (1, 29) = 30.43	**p<0.0001**		0.34
				Sham	2.281	1.668							treatment	F (1, 29) = 0.1001	p=0.7540		
			Frac 20 cGy: 16	Frac 20 cGy	2.768	1.293							subject	F (29, 29) = 0.8725	p=0.6421		
LDR training % reaching criteria	3B	CHOP (NSRL 18A)	Sham: 15	LD train criteria completion curve								Log-rank (Mantel-Cox) test	NA	NA	p=0.8480	NA	
				Sham	Median: 5												
			Frac 20 cGy: 16	Frac 20 cGy	Median: 9												
LDR training Days to completion	3C	CHOP (NSRL 18A)	Sham: 15	Days to completion								Unpaired t-test	NA	NA	p=0.9258	NA	
				Sham	6.133 ± 2.004												
			Frac 20 cGy: 16	Frac 20 cGy	5.938 ± 0.7386												

LDR training Trial number	3D	CHOP (NSRL 18A)	Sham: 15	Trial #				Two-way RM ANOVA	interaction	F (1, 29) = 0.4779	p=0.4949	NA	
				Session	First	Last			session	F (1, 29) = 1.549	p=0.2233		
				Sham	39.67	40.67			treatment	F (1, 29) = 0.001294	p=0.9716		
			Frac 20 cGy: 16	Frac 20 cGy	38.31	41.81			subject**	F (29, 29) = 2.566	p=0.0067		
LDR training Percent correct to 1st reversal	3E	CHOP (NSRL 18A)	Sham: 15	% correct				Two-way RM ANOVA	interaction	F (1, 29) = 8.180e-006	p=0.9977	NA	
				Session	First	Last			session	F (1, 29) = 0.9060	p=0.3490		
				Sham	65.91	70.49			treatment	F (1, 29) = 3.713	p=0.0638		
			Frac 20 cGy: 16	Frac 20 cGy	55.59	60.19			subject	F (29, 29) = 1.230	p=0.2905		
LDR test Large separation session length	3G	CHOP (NSRL 18A)	Sham: 15	Large separation session length (s)				Two-way RM ANOVA	interaction	F (2, 58) = 0.002083	p=0.9979	NA	
				Block	1	4	6		block	F (2, 58) = 2.002	p=0.1443		
				Sham	1800	1800	1798		treatment	F (1, 29) = 0.002083	p=0.9639		
			Frac 20 cGy: 16	Frac 20 cGy	1800	1800	1799		subject	F (29, 58) = 1.000	p=0.4857		
LDR test Small separation session length	3H	CHOP (NSRL 18A)	Sham: 15	Small separation session length (s)				Two-way RM ANOVA	interaction	F (2, 58) = 0.002083	p=0.9979	NA	
				Block	1	4	6		block	F (2, 58) = 2.002	p=0.1443		
				Sham	1800	1800	1800		treatment	F (1, 29) = 0.002083	p=0.9639		
			Frac 20 cGy: 16	Frac 20 cGy	1800	1800	1800		subject	F (29, 58) = 1.000	p=0.4857		
LDR test Large separation trial #	3I	CHOP (NSRL 18A)	Sham: 15	Large separation trial #				Two-way RM ANOVA	interaction	F (2, 58) = 1.877	p=0.1622	NA	
				Block	1	4	6		block	F (2, 58) = 1.737	p=0.1851		
				Sham	43.2	36.67	40.8		treatment	F (1, 29) = 0.4793	p=0.4943		
			Frac 20 cGy: 16	Frac 20 cGy	39.69	41.75	49.75		subject****	F (29, 58) = 3.552	p<0.0001		
LDR test Small separation trial #	3J	CHOP (NSRL 18A)	Sham: 15	Small separation trial #				Two-way RM ANOVA	interaction	F (2, 58) = 0.07135	p=0.9312	NA	
				Block	1	4	6		block	F (2, 58) = 2.175	p=0.1228		
				Sham	33.87	35.87	39.33		treatment	F (1, 29) = 1.916	p=0.1768		
			Frac 20 cGy: 16	Frac 20 cGy	39.31	42.88	44.56		subject****	F (29, 58) = 4.118	p<0.0001		
LDR test Large separation percent correct to 1st reversal	3K	CHOP (NSRL 18A)	Sham: 15	Large separation % correct to 1st reversal				Two-way RM ANOVA	interaction*	F (2, 58) = 3.761	**p=0.0291**		0.05
				Block	1	4	6		block	F (2, 58) = 0.5836	p=0.5611	Sham vs. 56Fe: *a'* p=0.0220	
				Sham	63.84	56.55	51.69		treatment	F (1, 29) = 1.230	p=0.2765		
			Frac 20 cGy: 16	Frac 20 cGy	58.97	57.54	69.31		subject	F (29, 58) = 1.413	p=0.1308		
LDR test Small separation percent correct to 1st reversal	3L	CHOP (NSRL 18A)	Sham: 15	Small separation % correct to 1st reversal				Two-way RM ANOVA	interaction	F (2, 58) = 1.149	p=0.3242	NA	
				Block	1	4	6		block	F (2, 58) = 0.5919	p=0.5566		
				Sham	40.99	50.29	38.53		treatment	F (1, 29) = 2.469	p=0.1269		
			Frac 20 cGy: 16	Frac 20 cGy	48.89	48.97	51.79		subject	F (29, 58) = 1.124	p=0.3452		
LDR test Large separation reversal #	3M	CHOP (NSRL 18A)	Sham: 15	Large separation reversal #				Two-way RM ANOVA	interaction	F (2, 58) = 1.034	p=0.3619	NA	
				Block	1	4	6		block	F (2, 58) = 2.559	p=0.0861		
				Sham	0.8667	1.267	0.9333		treatment	F (1, 29) = 0.1469	p=0.7043		
			Frac 20 cGy: 16	Frac 20 cGy	0.6875	1.25	1.375		subject	F (29, 58) = 1.369	p=0.1537		
LDR test Small separation reversal #	3N	CHOP (NSRL 18A)	Sham: 15	Small separation reversal #				Two-way RM ANOVA	interaction	F (2, 58) = 0.03173	p=0.9688	NA	
				Block	1	4	6		block	F (2, 58) = 1.992	p=0.1457		
				Sham	0.2	0.6	0.3333		treatment	F (1, 29) = 1.777	p=0.1928		
			Frac 20 cGy: 16	Frac 20 cGy	0.5	0.8125	0.5625		subject	F (29, 58) = 1.516	p=0.0888		
LDR test Large separation blank touch	3O	CHOP (NSRL 18A)	Sham: 15	Large separation blank touch				Two-way RM ANOVA	interaction	F (2, 58) = 0.9088	p=0.4087		
				Block	1	4	6		block	F (2, 58) = 0.7784	p=0.4639	Sham vs. 56Fe: *a'* p=0.0232	
				Sham	55.6	55.27	54.93		treatment*	F (1, 29) = 6.262	**p=0.0182**		0.09
			Frac 20 cGy: 16	Frac 20 cGy	67.81	66.06	78.69		subject**	F (29, 58) = 2.094	p=0.0084		
LDR test Small separation blank touch	3P	CHOP (NSRL 18A)	Sham: 15	Small separation blank touch				Two-way RM ANOVA	interaction	F (2, 58) = 0.1156	p=0.8910		
				Block	1	4	6		block	F (2, 58) = 2.185	p=0.1216	Sham vs 56Fe: *a'* p=0.0204 Block 4, *a'* p=0.0292 Block 6	
				Sham	63.33	48.07	59		treatment**	F (1, 29) = 11.49	**p=0.0020**		0.17
			Frac 20 cGy: 16	Frac 20 cGy	88.25	78.81	88.31		subject***	F (29, 58) = 2.625	p=0.0009		
LDR test Large separation reward latency	3Q	CHOP (NSRL 18A)	Sham: 15	Large separation reward latency (s)				Two-way RM ANOVA	interaction	F (2, 58) = 1.095	p=0.3414	NA	
				Block	1	4	6		block	F (2, 58) = 0.6710	p=0.5151		
				Sham	0.7607	0.97	0.878		treatment	F (1, 29) = 1.083	p=0.3066		
			Frac 20 cGy: 16	Frac 20 cGy	2.284	0.8806	0.9556		subject	F (29, 58) = 0.9787	p=0.5122		
LDR test Small separation reward latency	3R	CHOP (NSRL 18A)	Sham: 15	Small separation reward latency (s)				Two-way RM ANOVA	interaction	F (2, 58) = 1.426	p=0.2485	NA	
				Block	1	4	6		block	F (2, 58) = 1.675	p=0.1962		
				Sham	0.6313	0.5907	0.5373		treatment	F (1, 29) = 1.971	p=0.1709		
			Frac 20 cGy: 16	Frac 20 cGy	2.311	0.6363	0.6406		subject	F (29, 58) = 0.9379	p=0.5642		
LDR test Large separation correct image latency	3S	CHOP (NSRL 18A)	Sham: 15	Large separation correct image latency (s)				Two-way RM ANOVA	interaction	F (2, 58) = 0.9595	p=0.3891	NA	
				Block	1	4	6		block	F (2, 58) = 1.298	p=0.2808		
				Sham	8.746	23.89	11.76		treatment	F (1, 29) = 0.2292	p=0.6357		
			Frac 20 cGy: 16	Frac 20 cGy	7.699	12.25	17.06		subject	F (29, 58) = 1.040	p=0.4380		
LDR test Small separation correct image latency	3T	CHOP (NSRL 18A)	Sham: 15	Small separation correct image latency (s)				Two-way RM ANOVA	interaction	F (2, 58) = 0.8020	p=0.4533		
				Block	1	4	6		block	F (2, 58) = 1.979	p=0.1474	Sham vs. 56Fe: all p>0.05	
				Sham	26.37	17.31	12.21		treatment*	F (1, 29) = 4.517	**p=0.0422**		0.04
			Frac 20 cGy: 16	Frac 20 cGy	12.11	9.445	9.084		subject	F (29, 58) = 1.208	p=0.2655		
LDR test Large separation incorrect image latency	3U	CHOP (NSRL 18A)	Sham: 15	Large separation incorrect image latency (s)				Two-way RM ANOVA	interaction	F (2, 58) = 0.4078	p=0.6670	NA	
				Block	1	4	6		block	F (2, 58) = 1.628	p=0.2051		
				Sham	6.914	15.63	12.22		treatment	F (1, 29) = 1.640	p=0.2105		
			Frac 20 cGy: 16	Frac 20 cGy	5.971	8.786	9.316		subject	F (29, 58) = 1.050	p=0.4259		
LDR test Small separation incorrect image latency	3V	CHOP (NSRL 18A)	Sham: 15	Small separation incorrect image latency (s)				Two-way RM ANOVA	interaction	F (2, 58) = 0.6914	p=0.5049	NA	
				Block	1	4	6		block	F (2, 58) = 0.3911	p=0.6781		
				Sham	12.58	13.54	12.93		treatment	F (1, 29) = 0.4058	p=0.5291		
			Frac 20 cGy: 16	Frac 20 cGy	14.63	8.693	10.21		subject	F (29, 58) = 1.391	p=0.1417		

Acquisition Criteria completion curve	4B	CHOP (NSRL 18A)	Sham: 16<br>Frac 20 cGy: 16	Acquisition criteria completion curve<br>Sham Median: 27<br>Frac 20 cGy Median: 37	Log-rank (Mantel-Cox) test	NA	NA	p>0.9999	NA	
Acquisition Days to completion	4C	CHOP (NSRL 18A)	Sham: 16<br>Frac 20 cGy: 16	Days to completion<br>Sham 19.56 ± 4.460<br>Frac 20 cGy 20.31 ± 4.534	Unpaired t-test	NA	NA	p=0.9069	NA	
Acquisition Session length	4D	CHOP (NSRL 18A)	Sham: 15<br>Frac 20 cGy: 15	Session length (s)<br>Session / First / Last<br>Sham / 1285 / 813.3<br>Frac 20 cGy / 1187 / 760.6	Two-way RM ANOVA	interaction<br>session***<br>treatment<br>subject**	F (1, 28) = 0.1246<br>F (1, 28) = 50.16<br>F (1, 28) = 0.4781<br>F (28, 28) = 2.939	p=0.7267<br>p<0.0001<br>p=0.4950<br>p=0.0029	NA	
Acquisition Correct response #	4E	CHOP (NSRL 18A)	Sham: 15<br>Frac 20 cGy: 15	Correct reponse #<br>Session / First / Last<br>Sham / 27.47 / 29.87<br>Frac 20 cGy / 29.6 / 30	Two-way RM ANOVA	interaction<br>session<br>treatment<br>subject	F (1, 28) = 2.073<br>F (1, 28) = 4.063<br>F (1, 28) = 2.071<br>F (28, 28) = 1.286	p=0.1610<br>p=0.0535<br>p=0.1612<br>p=0.2553	NA	
Ext Criteria completion curve	4G	CHOP (NSRL 18A)	Sham: 15<br>Frac 20 cGy: 15	Extinction criteria completion curve<br>Sham Median: 18<br>Frac 20 cGy Median: 23	Log-rank (Mantel-Cox) test	NA	NA	p=0.6830	NA	
Ext Days to completion	4H	CHOP (NSRL 18A)	Sham: 15<br>Frac 20 cGy: 15	Days to completion<br>Sham 16.21 ± 1.867<br>Frac 20 cGy 13.58 ± 1.574	Unpaired t-test	NA	NA	p=0.3012	NA	
Ext Session length	4I	CHOP (NSRL 18A)	Sham: 13<br>Frac 20 cGy: 14	Session length (s)<br>Session / 1 / 8 / Last<br>Sham / 506.2 / 567.5 / 580.9<br>Frac 20 cGy / 513 / 571.0 / 590.7	Two-way RM ANOVA	interaction<br>session****<br>treatment<br>subject	F (2, 50) = 0.1365<br>F (2, 50) = 87.44<br>F (1, 25) = 1.077<br>F (25, 50) = 1.709	p=0.8727<br>**p<0.0001**<br>p=0.3092<br>p=0.0533	NA	0.64
Ext Omission #	4J	CHOP (NSRL 18A)	Sham: 13<br>Frac 20 cGy: 14	Omission #<br>Session / 1 / 6 / 11 / 16 / 21<br>Sham / 12 / 20.43 / 22.64 / 24.71 / 24.03<br>Frac 20 cGy / 12 / 20 / 22.38 / 24.15 / 24.7	Mixed-effects analysis	day of test****<br>treatment<br>interaction	F (23, 559) = 30.96<br>F (1, 25) = 0.05308<br>F (23, 559) = 1.101	**p<0.0001**<br>p=0.8197<br>p=0.3389	NA	
Ext Blank touch	4K	CHOP (NSRL 18A)	Sham: 13<br>Frac 20 cGy: 14	Blank touch<br>Session / 1 / 8 / Last<br>Sham / 14.85 / 17.08 / 16.08<br>Frac 20 cGy / 20.21 / 22.57 / 20.64	Two-way RM ANOVA	interaction<br>session<br>treatment<br>subject**	F (2, 50) = 0.02480<br>F (2, 50) = 0.5277<br>F (1, 25) = 2.831<br>F (25, 50) = 2.741	p=0.9755<br>p=0.5932<br>p=0.1049<br>p=0.0012	NA	
Ext Blank touch latency	4L	CHOP (NSRL 18A)	Sham: 13<br>Frac 20 cGy: 14	Blank touch latency (s)<br>Session / 1 / 8 / Last<br>Sham / 2.882 / 3.818 / 4.569<br>Frac 20 cGy / 3.145 / 3.706 / 3.926	Two-way RM ANOVA	interaction<br>session***<br>treatment<br>subject	F (2, 50) = 1.324<br>F (2, 50) = 9.867<br>F (1, 25) = 0.3933<br>F (25, 50) = 1.314	p=0.2752<br>**p=0.0002**<br>p=0.5363<br>p=0.2024	NA	0.17
Ext ITI touch	4M	CHOP (NSRL 18A)	Sham: 13<br>Frac 20 cGy: 14	ITI Touch<br>Session / 1 / 8 / Last<br>Sham / 48.15 / 37.85 / 31.08<br>Frac 20 cGy / 45.93 / 38.64 / 35.83	Two-way RM ANOVA	interaction<br>session***<br>treatment<br>subject**	F (2, 50) = 0.5152<br>F (2, 50) = 7.996<br>F (1, 25) = 0.06728<br>F (25, 50) = 2.302	p=0.6005<br>**p=0.0010**<br>p=0.7975<br>p=0.0060	NA	0.11
Ext Response latency	4N	CHOP (NSRL 18A)	Sham: 13<br>Frac 20 cGy: 14	Response latency (s)<br>Session / 1 / 8 / Last<br>Sham / 4.54 / 5.150 / 4.753<br>Frac 20 cGy / 4.431 / 4.628 / 6.302	Two-way RM ANOVA	interaction*<br>session*<br>treatment<br>subject	F (2, 50) = 4.173<br>F (2, 50) = 3.835<br>F (1, 25) = 1.512<br>F (25, 50) = 0.6457	**p=0.0211**<br>**p=0.0282**<br>p=0.2302<br>p=0.8813	Sham vs. 56Fe: *a'* p=0.0088	0.08<br>0.08
General Touchscreen Training w/three windows	5A	UTSW (NSRL 17B)	Sham: 11<br>Frac 20 cGy: 9	General TS training<br>Training Stage / Hab 2 / IT / MT / MI / PI<br>Sham / 1.273 / 1.182 / 4.909 / 1.273 / 12<br>Frac 20 cGy / 1.222 / 1 / 4.222 / 2.873 / 10.89	Mixed-effects analysis	training phase****<br>treatment<br>interaction	F (4, 71) = 116.1<br>F (1, 18) = 0.04624<br>F (4, 71) = 1.573	**p<0.0001**<br>p=0.8322<br>p=0.1908	NA	
VMCL train/test	5D	UTSW (NSRL 17B)	Sham: 11<br>Frac 20 cGy: 9	VMCL train/test<br>Experiment phase / Train / Test<br>Sham / 26.27 / 26.73<br>Frac 20 cGy / 25 / 42.22	Mixed-effects analysis	experiment phase*<br>treatment*<br>interaction*	F (1, 18) = 8.145<br>F (1, 18) = 6.334<br>F (1, 18) = 7.329	**p=0.0105**<br>**p=0.0215**<br>**p=0.0144**	Sham vs. 56Fe: *a'* p=0.0014	0.15<br>0.11
VMCL train criteria completion curve	5E	UTSW (NSRL 17B)	Sham: 11<br>Frac 20 cGy: 9	VMCL train criteria completion curve<br>Sham Median: 31<br>Frac 20 cGy Median: 31	Log-rank (Mantel-Cox) test	NA	NA	p=0.6512	NA	
VMCL train Session length	5F	UTSW (NSRL 17B)	Sham: 11<br>Frac 20 cGy: 9	Session length (s)<br>Session / First / Last<br>Sham / 1800 / 1425<br>Frac 20 cGy / 1800 / 1587	Two-way RM ANOVA	interaction<br>session****<br>treatment<br>subject	F (1, 18) = 3.606<br>F (1, 18) = 47.84<br>F (1, 18) = 3.606<br>F (18, 18) = 1.000	p=0.0737<br>**p<0.0001**<br>p=0.0737<br>p=0.5000	NA	0.55
VMCL train Trial #	5G	UTSW (NSRL 17B)	Sham: 11<br>Frac 20 cGy: 9	Trial #<br>Session / First / Last<br>Sham / 12.91 / 25<br>Frac 20 cGy / 15.22 / 24.67	Two-way RM ANOVA	interaction<br>session****<br>treatment<br>subject	F (1, 18) = 1.629<br>F (1, 18) = 107.9<br>F (1, 18) = 1.034<br>F (18, 18) = 0.8817	p=0.2181<br>**p<0.0001**<br>p=0.3227<br>p=0.6039	NA	0.55
VMCL train Percent correct	5H	UTSW (NSRL 17B)	Sham: 11<br>Frac 20 cGy: 9	% correct<br>Session / First / Last<br>Sham / 77.68 / 91.27<br>Frac 20 cGy / 71.99 / 90.14	Two-way RM ANOVA	interaction<br>session***<br>treatment<br>subject	F (1, 18) = 0.4270<br>F (1, 18) = 20.66<br>F (1, 18) = 1.543<br>F (18, 18) = 0.6181	p=0.5217<br>**p=0.0003**<br>p=0.2302<br>p=0.8417	NA	-0.01
VMCL train Correction trial	5I	UTSW	Sham: 11	Correction trial #<br>Session / First / Last	Two-way RM	interaction<br>session**	F (1, 18) = 1.611<br>F (1, 18) = 9.162	p=0.2205<br>**p=0.0072**	NA	0.19

Correction trial #	5I	(NSRL 17B)		Sham	3.909		2.545		ANOVA	treatment	F (1, 18) = 1.928	p=0.1819	NA	
			Frac 20 cGy: 9	Frac 20 cGy	5.889		2.556			subject	F (18, 18) = 0.8527	p=0.6305		
VMCL test Criteria completion curve	5J	UTSW (NSRL 17B)	Sham: 11			VMCL test criteria completion curve			Log-rank (Mantel-Cox) test	NA	NA	p = 0.6501	NA	
				Sham		Median: 36								
			Frac 20 cGy: 9	Frac 20 cGy		Median: 47								
VMCL test Session length	5K	UTSW (NSRL 17B)	Sham: 11			Session length (s)			Mixed-effects analysis	session	F (3, 69) = 1.318	p=0.2755	NA	
				Session	1	8	13	22						
				Sham	1800	1800	1754	1737		treatment	F (1, 69) = 2.212	p=0.1415		
			Frac 20 cGy: 9	Frac 20 cGy	1800	1800	1800	1786		interaction	F (3, 69) = 0.7373	p=0.5334		
VMCL test Percent correct	5L	UTSW (NSRL 17B)	Sham: 11			% correct			Mixed-effects analysis	session	F (3, 51) = 1.878	p=0.1450	NA	
				Session	1	8	13	22						
				Sham	40.57	49.59	56.33	61.52		treatment	F (1, 18) = 0.4497	p=0.5110		
			Frac 20 cGy: 9	Frac 20 cGy	50	36.7	50.05	54.53		interaction	F (3, 51) = 0.9603	p=0.4186		
VMCL test Percent missed	5M	UTSW (NSRL 17B)	Sham: 11			% missed			Mixed-effects analysis	session	F (3, 51) = 0.9020	p=0.4467	NA	
				Session	1	8	13	22						
				Sham	30.63	19.78	21.79	17.08		treatment	F (1, 18) = 0.2195	p=0.6451		
			Frac 20 cGy: 9	Frac 20 cGy	26.36	27.05	26.95	19.42		interaction	F (3, 51) = 0.3523	p=0.7876		
VMCL test Incorrect trial #	5N	UTSW (NSRL 17B)	Sham: 11			Incorrect trial #			Mixed-effects analysis	session	F (3, 51) = 1.016	p=0.3931	NA	
				Session	1	8	13	22						
				Sham	4	3.182	4.182	4.657		treatment	F (1, 18) = 0.04586	p=0.8328		
			Frac 20 cGy: 9	Frac 20 cGy	4	4.222	3.667	4.778		interaction	F (3, 51) = 0.6279	p=0.6003		
Elevated Plus Maze Open arm time	6B	CHOP (NSRL 18A)	Sham: 14			Open arm time (s)			Unpaired t-test	NA	NA	p=0.2052	NA	
				Sham		54.48 ± 4.561								
			Frac 20 cGy: 15	Frac 20 cGy		71.07 ± 11.58								
Elevated Plus Maze Closed arm time	6C	CHOP (NSRL 18A)	Sham: 14			Closed arm time (s)			Unpaired t-test	NA	NA	p=0.2298	NA	
				Sham		192.1 ± 5.199								
			Frac 20 cGy: 15	Frac 20 cGy		177.8 ± 10.20								
Marble Burying Percent marbles	6D	CHOP (NSRL 18A)	Sham: 14			% marbles buried			Unpaired t-test	NA	NA	p=0.2729	NA	
				Sham		26.25 ± 5.161								
			Frac 20 cGy: 15	Frac 20 cGy		34.17 ± 4.847								
Open Field Distance moved	6E	CHOP (NSRL 18A)	Sham: 14			Distance moved (cm)			Unpaired t-test	NA	NA	p=0.5218	NA	
				Sham		2589 ± 205.3								
			Frac 20 cGy: 15	Frac 20 cGy		2411 ± 180.9								
Open Field Center area time	6F	CHOP (NSRL 18A)	Sham: 14			Center area time (s)			Unpaired t-test	NA	NA	p=0.1958	NA	
				Sham		12.50 ± 2.163								
			Frac 20 cGy: 15	Frac 20 cGy		9.203 ± 1.303								
Open Field Corner area time	6G	CHOP (NSRL 18A)	Sham: 14			Corner area time (s)			Unpaired t-test	NA	NA	p=0.5020	NA	
				Sham		24.90 ± 4.424								
			Frac 20 cGy: 15	Frac 20 cGy		21.14 ± 3.376								
Social interaction Interaction time	6H	CHOP (NSRL 18A)	Sham: 14			Interaction time (s)			Two-way RM ANOVA	interaction	F (1, 27) = 0.3585	p=0.5543	NA	0.15
				Target presence	without		with			target****	F (1, 27) = 21.43	**p<0.0001**		
				Sham	75.46		89.91			treatment	F (1, 27) = 0.8003	p=0.3789		
			Frac 20 cGy: 15	Frac 20 cGy	79.05		97.79			subject**	F (27, 27) = 3.200	p=0.0018		
Forced Swim Test Immobile time	6I	CHOP (NSRL 18A)	Sham: 14			Immobile time (s)			Unpaired t-test	NA	NA	p=0.7936	NA	
				Sham		65.72 ± 14.20								
			Frac 20 cGy: 15	Frac 20 cGy		70.51 ± 11.47								
